# Oxidant/Antioxidant Profile in the Thoracic Aneurysm of Patients with the Loeys-Dietz Syndrome

**DOI:** 10.1155/2020/5392454

**Published:** 2020-03-23

**Authors:** Maria Elena Soto, Lináloe G. Manzano-Pech, Verónica Guarner-Lans, Jorge A. Díaz-Galindo, Xicoténcatl Vásquez, Vicente Castrejón-Tellez, Ricardo Gamboa, Claudia Huesca, Giovanny Fuentevilla-Alvárez, Israel Pérez-Torres

**Affiliations:** ^1^Immunology Department, Instituto Nacional de Cardiología “Ignacio Chávez”, Juan Badiano 1, Sección XVI, Tlalpan, 14080 México City, Mexico; ^2^Cardiovascular Biomedicine Department, Instituto Nacional de Cardiología “Ignacio Chávez”, Juan Badiano 1, Sección XVI, Tlalpan, 14080 México City, Mexico; ^3^Physiology Department, Instituto Nacional de Cardiología “Ignacio Chávez”, Juan Badiano 1, Sección XVI, Tlalpan, 14080 México City, Mexico; ^4^Cardiothoracic Surgery Department, Instituto Nacional de Cardiología “Ignacio Chávez”, Juan Badiano 1, Sección XVI, Tlalpan, 14080 México City, Mexico

## Abstract

Patients with the Loeys-Dietz syndrome (LDS) have mutations in the TGF-*β*R1, TGF-*β*R2, and SMAD3 genes. However, little is known about the redox homeostasis in the thoracic aortic aneurysms (TAA) they develop. Here, we evaluate the oxidant/antioxidant profile in the TAA tissue from LDS patients and compare it with that in nondamaged aortic tissue from control (C) subjects. We evaluate the enzymatic activities of glutathione peroxidase (GPx), glutathione S-transferase (GST), glutathione reductase (GR), catalase (CAT), superoxide dismutase (SOD) isoforms, and thioredoxin reductase (TrxR). We also analyze some antioxidants from a nonenzymatic system such as selenium (Se), glutathione (GSH), and total antioxidant capacity (TAC). Oxidative stress markers such as lipid peroxidation and carbonylation, as well as xanthine oxidase (ORX) and nuclear factor erythroid 2-related factor 2 (Nrf2) expressions, were also evaluated. TAA from LDS patients showed a decrease in GSH, Se, TAC, GPx, GST, CAT, and TrxR. The SOD activity and ORX expressions were increased, but the Nrf2 expression was decreased. The results suggest that the redox homeostasis is altered in the TAA from LDS patients, favoring ROS overproduction that contributes to the decrease in GSH and TAC and leads to LPO and carbonylation. The decrease in Se and Nrf2 alters the activity and/or expression of some antioxidant enzymes, thus favoring a positive feedback oxidative background that contributes to the TAA formation.

## 1. Introduction

The Loeys-Dietz syndrome (LDS) is a variant of the Marfan syndrome (MS), and in it, as in all complex genetic diseases, there is damage to different organs and systems. Bart Loeys and Harry Dietz described the LDS in 2005 [1]. This syndrome is an autosomal dominant disease due to mutations in several genes including the gene that encodes for the beta I receptor of transforming growth factor (TGF-*β*R1), which is located on chromosome 9q22.33; mutations in the TGF-*β*R2 gene, located on chromosome 3p24.1; and mutations in the SMAD3 gene located on chromosome 15q22.33. The LDS is characterized by unusual facies, dolichocephaly, craniosynostosis, hypertelorism, malar hypoplasia, arched palate, wide or bifid uvula, retrognathia, sternum deformity, scoliosis, joint laxity, dural ectasia, long fingers without bone growth, contractures, equine foot, translucent skin, and learning problems [1]. The patients with LDS show complications during pregnancy, such as rupture of the uterus and death [2]. The patients also have a strong predisposition to arterial tortuosity [3] and a high frequency in the appearance of thoracic aortic aneurysms (TAA) at an early age [4]. The TAA in LDS are characterized by a high risk of dissection or rupture, even when diameters are small (less than 5 cm). Up to 53% of patients also develop aneurysms in other aortic locations [5]. The first histological alterations described in TAA from LDS showed similarities with lesions in aortas from MS. In both syndromes, a loss of content of elastic fibers and their disordered disposition were found [5]. There was also an increase in the collagen deposition. However, in LDS, these changes are more aggressive than those in MS [6], resulting in a faster growth of the aneurysm (growing rate of 1.0 cm/year), that may lead to premature death [5]. At present, 5 different types of LDS have been reported each having different clinical characteristics. For example, the characteristics of type 2 LDS may not be easily recognized from those of MS, since in this form of LDS, there are some musculoskeletal criteria similar to those of MS that can be easily confused or lead to a late diagnosis of the disease [7].

In the development of the TAA in MS and other related diseases, there is a presence of endothelial dysfunction (ED) and oxidative stress (OS) [8]. The endothelium directly responds to the physical forces of blood flow losing the inflammatory/anti-inflammatory balance and leading to the release of mediators that participate in the regulation of the vascular tone. As an important part of this imbalance, there are alterations in the NO produced by eNOS [9].

Regarding OS, overproduction of ROS can contribute to arterial wall degeneration [9–11], as has been described in animal models and in humans with MS [12, 13]. ROS production is linked to ED. When the substrate in the synthesis of the NO is insufficient, the transfer of electrons is diverted from O_2_ to highly reactive substances such as superoxide (O_2_^−^) and hydrogen peroxide (H_2_O_2_). These products damage the lipids of the cell membrane, the proteins, and the DNA. They also inactivate antioxidant enzymes, increase the proinflammatory gene expression, and induce cell damage and apoptosis [14]. However, not only do reactive oxygen species (ROS) come from the NO synthesis pathway but also they originated from xanthine oxidase (ORX), NADPH dehydrogenase, cytochrome p450, and the mitochondria.

Antioxidant systems are also present in cells, and they constitute a compensatory mechanism, which are activated to reduce the damage caused by ROS and are capable of stabilizing and neutralizing the oxidative effects [15]. These antioxidant systems are divided into the enzymatic and nonenzymatic systems. Superoxide dismutase (SOD) isoforms, glutathione S-transferase (GST), catalase (CAT), glutathione reductase (GR), glutathione peroxidase (GPx), and thioredoxin reductase (TrxR) form part of the enzymatic antioxidant system [14]. The nonenzymatic system includes antioxidant substances such as selenium (Se), vitamins E and C, and the glutathione tripeptide (GSH) among others [15].

In studies on the role of OS in MS, the enzymatic and nonenzymatic systems show disequilibrium [14]. The activities of the enzymes that use GSH are decreased [14], and iNOS expression and the nitrate and nitrite ratio (NO_3_^−^/NO_2_^−^) are increased. These changes are associated with a decrease in the total antioxidant capacity (TAC) and an increase in LPO and carbonylation [16]. These changes contribute to a chronic inflammatory process [10], which may lead to the development of the aneurysm in the aortic root [17, 18]. Although there are several studies on the role of OS in TAA in MS, there are no reports showing the participation or presence of OS in the development of TAA in LDS. Therefore, the goal of this investigation was to analyze the oxidant/antioxidant profile in the TAA of LDS patients and to assess the participation of OS in this syndrome.

## 2. Material and Methods

### 2.1. Ethical Considerations

The research protocol was approved by the research and ethics committee of the National Institute of Cardiology “Ignacio Chávez” (protocol number 09654). An informed consent form was presented and signed by each LDS patient and control individual in accordance with the Helsinki declaration, as amended by the congress of Tokyo, Japan [19]. This was an observational and comparative study that was performed in a prospective cohort of patients that attended our institution and that were assisted to the aorta clinic of the National Institute of Cardiology “Ignacio Chávez” between March 2011 and March 2019.

Based on the inclusion criteria, 4 men with ages ranging from 13 to 46 years and 6 women with ages between 14 and 35 years were included. The patients with LDS were incorporated when they had been evaluated and diagnosed by an expert rheumatologist (M.E.S.) according to the criteria proposed by Ghent [20]. When the patients had other characteristics that suggested a diagnosis more of LDS than of MS, genetic tests were performed to unmask mutations in the FBN-1, TGF-BR2, and COL3A1 genes. In patients in whom LDS was diagnosed, there were hyperelasticity and hypermobility similar to those in the Ehlers-Danlos syndrome. However, nowadays there is no international consensus on diagnostic criteria of LDS. Patients were programmed for surgery, when they had ≥4.5 cm dilation, shown by magnetic resonance or computerized tomography, and had been previously presented and discussed in an interdisciplinary meeting with surgeons and the heart team (HT) or when they had attended the institute for the first time with aortic dissection and/or dilation. The surgical techniques employed were Bentall and Bono and David type 5 according to their arterial complication and the final decision by the HT [21].

The control (C) subjects included had no aortic damage and did not undergo surgery for aortic stenosis, having had no syndrome pathology diagnosed. In these patients, there was no suspicion of inflammatory disease or presence of degenerative disorders such as thyroid and autoimmune diseases, diabetes mellitus, and arterial hypertension. Medications that could interfere with the outcome of the study such as lipid-lowering drugs or NSAIDs were suspended in the perioperative period. Cases were dealt with caution, to avoid including patients undertaking treatment with allopurinol, antioxidants, or probable inhibitors of ROS production pathways. Warfarin, aspirin, clopidogrel, anticoagulant, and antiplatelet medications and other drugs were suspended.

In patients with LDS and C subjects, laboratory tests were made to determine acute-phase reactants, high-density lipoprotein (HDL), low-density lipoprotein (LDL), cholesterol (CT), and triglycerides (TG). Additionally, image studies by magnetic resonance echocardiography or computerized tomography were done to discard aortic damage in addition to valvular damage. We assessed the presence of congenital heart anomalies (CHA) including tetralogy of Fallot, atrial septal defect, patent ductus arteriosus, or bicuspid aortic valve based on echocardiographic images or institutional MR angiographies because in these patients, the CHA are related and support the clinical diagnosis.

Based on the exclusion criteria, we excluded two patients because the integral genetic study was not possible.

The Bentall technique consists in the replacement of the aortic valve root and ascending aorta with a Dacron tube, to which both coronary arteries are anastomosed on the lateral faces and at one of its ends to a valvular prosthesis. The difference between the Bentall technique and the David type 5 techniques is that in the latter, the native aortic valve and valve commissures are preserved and are reimplanted within the Dacron tube [21].

A segment of the TAA was taken on the surgery; the tissue was placed in LN_2_ and was kept at -70°C until used.

### 2.2. Thoracic Aortic Aneurysm Tissue Homogenization

The segment from the TAA was homogenized in LN_2_, according to the methods that were previously described by Soto et al. [16]. The Bradford method was utilized to determine the protein concentration in the homogenates [22]. All assays on biochemical variables (except for those in the native gels and western blot analysis) were made in duplicate.

### 2.3. Superoxide Dismutase and Catalase Activities in Native Gels

The measurement of the activity of the SOD isoforms and CAT was performed by electrophoresis in 10% polyacrylamide native gels. CAT and SOD isoforms were revealed according to the methods that were previously described by Pérez-Torres et al. [23]. Purified SOD from bovine erythrocytes with a specific activity of 112 U/mg of protein (Sigma-Aldrich, St. Louis, MO, USA) and purified CAT from a bovine liver having a specific activity of 60 U/mg (Sigma-Aldrich) were used as positive controls. The previously mentioned antioxidant enzyme activity determinations were performed according to the manufacturer's instructions. Samples were placed in a separate lane of the gel and run in parallel with the biological samples. The intensity of the signal from the controls was used as a reference to measure the enzymatic activity in the tissue samples. Therefore, the results are expressed as U of activity per mg of protein. The gels were analyzed by densitometry with image SigmaScan Pro 5.1 software (Systat Software, Inc., San Jose, CA, USA).

### 2.4. Enzymes That Use Glutathione and/or Oxidized Glutathione

For the GPx, GST, and GR activities, 100 *μ*g of the protein of the TAA homogenate was utilized according to previously described methods [24, 25]. The GPx activity is expressed as nmol of NADPH oxidized/min/mg protein, with an extinction coefficient of 6220 M^–1^ cm^–1^ at 340 nm for NADPH. The GST activity is expressed as units of GS-TNB mol/min/mg protein with an extinction coefficient of 14150 M^−1^ cm^−1^. The GR activity is expressed as *μ*mol of reduced GSSG/min/mg protein, with an extinction coefficient of 6220 M^–1^ cm^–1^.

### 2.5. Thioredoxin Reductase

The TrxR activity was determined indirectly by the amount of DTNB in the presence of NADPH to form 2 moles of TNB. The DTNB oxidation is monitored at 412 nm at 37°C for 6 min with an extinction coefficient of 13600 M^−1^ cm^−1^. 100 *μ*g of TAA homogenate protein suspended in 3 ml of 0.1 mM phosphate buffer (KH_2_PO_4_, pH 7.0) was added to 0.2 mM NADPH, 1 mM EDTA, and 0.1 mg/ml bovine serum albumin. The samples were read in the presence of 20 *μ*l of the specific TrxR inhibitor (10 *μ*M auranofin) and together with a duplicate of the sample without the inhibitor [26].

### 2.6. Evaluation of Oxidative Stress Markers

For the measurement of the protein carbonylation, 1 mg of protein from the TAA homogenate was processed according to the method described by Pech et al. [27]. Absorbance was read at 370 nm, using air as the blank and a molar absorption coefficient of 22000 M^−1^ cm^−1^.

### 2.7. Lipid Peroxidation

1 mg of protein from the TAA homogenate was measured by the TBARS method that has been previously reported [23]. In this assay, the product malondialdehyde (MDA), which is a reactive aldehyde produced by lipid peroxidation (LPO) of polyunsaturated fatty acids that form a 1 : 2 adduct with thiobarbituric acid, is determined. The absorbance was read at 532 nm and is proportional to the concentration of MDA and the calibration curve obtained using tetraethoxypropane as the standard.

### 2.8. The Total Antioxidant Capacity

The total antioxidant capacity (TAC) of the nonenzymatic system was determined in 1 mg of protein from the TAA homogenate according to the method that was previously described by Benzie and Strain [28]. The absorbance was measured at 593 nm. The calibration curve was obtained using 6-hydroxy-2,5,7,8-tetramethylchroman-2-carboxylic acid, which is an analogue of vitamin E, and it is commonly used as a positive control in antioxidant assays.

### 2.9. Quantification of GSH Concentrations

In 1 mg of protein from the TAA homogenate, GSH levels were measured by the Ellman reagent (5,5-dithiobis-2-nitrobenzoic), according to the method described by Ellman [29]. The absorbance was read at 412 nm. The calibration curve was determined using GSH at concentrations ranging from 5 to 25 *μ*M.

### 2.10. Quantification of Vitamin C

1 mg of the protein from the TAA homogenate was mixed with C_2_HCl_3_O_2_ (20%) and was kept in an ice bath for 5 min and centrifuged at 5000 rpm for 5 min. The supernatant was recuperated and added to 200 *μ*l of Folin-Ciocalteu reagent (0.20 mM) and incubated for 10 min. The absorbance was measured at 760 nm [30].

### 2.11. Selenium

The technique used was previously described by Pérez Ruiz et al. [31], with some adaptation and modification described by Rodríguez et al. [32] and with some modifications and adaptations in our laboratory. All solutions were made with tridistilled H_2_O and are only used for the assay and discarded. In brief, in new Corning sterile polypropylene centrifuge tubes, 250 *μ*g of TAA homogenate protein and 500 *μ*l of acid mixture (4 : 1 vol/vol of HNO_3_+HCl) plus 500 *μ*l of 10% H_2_O_2_ were added and incubated in a sand bath at 120°C for 4 hours. After incubation, 100 *μ*l of tridistilled H_2_O, 150 *μ*l of 0.5 N NaOH, 200 *μ*l of 30% formaldehyde, 200 *μ*l of a mixture containing 0.5 N N_2_S and 0.5 N Na_2_SO_3_, plus 250 *μ*l of 0.01 M EDTA (pH 10.2), and 300 *μ*l of 4 mM toluidine blue were added. Samples were incubated for 15 min at 25°C. At the end of the incubation, they were centrifuged at 448 rcf for 2 min and the absorbance was read at 600 nm. The calibration curve was determined with 100 ng/ml Na_2_SeO_3_ which was treated under conditions similar to the experimental samples.

### 2.12. Western Blotting of eNOS, Nrf2, Cu/Zn-SOD, and ORX

50 *μ*g of protein from the homogenate was run on 12% SDS-PAGE (bis-acrylamide-laemmli gel) and blotted onto a polyvinylidene difluoride (PVDF) membrane (0.22 *μ*m, Millipore, Billerica, MA, USA) and then blocked 1 h at room temperature with Tris buffer solution-0.01% Tween (TBS-T 0.01%) plus 5% nonfat milk. The membranes were incubated overnight at 4°C with mouse primary monoclonal antibodies eNOS (sc-376751), Nrf2 (sc-365949), Cu/Zn-SOD (sc-101523), and ORX (sc-398548) from Santa Cruz Biotechnology, Santa Cruz, CA, USA, with 1 : 1000 dilution. After that, the membranes were incubated overnight at 4°C with a secondary antibody that is conjugated with horseradish peroxidase with 1 : 10000 dilutions (Santa Cruz Biotechnology, Santa Cruz, CA, USA). All the blots were incubated with an *α*-actin antibody as a load control. The protein was detected by the chemiluminescence assay (Clarity Western ECL Substrate, Bio-Rad Laboratories, Inc., Hercules, CA, USA). Chemiluminescence that was emitted in this process was detected in X-ray films (AGFA, Ortho CP-GU, Agfa HealthCare NV, Belgium). Images from each film were acquired with a GS-800 densitometer (including Quantity One software from Bio-Rad). The values of the density of each band are expressed as arbitrary units (AU).

### 2.13. Histology

A segment of 2 mm from the TAA was processed according to conventional histological techniques and stained with hematoxylin and eosin (HE) staining, Weigert's staining for elastic fibers, and Masson's trichrome staining. Histological sections were acquired according to the method and equipment described by Zúñiga-Muñoz et al. [14] and examined at a 40x magnification.

### 2.14. Assay Design and the HRMA Technique

High-resolution melting analysis (HRMA) was performed on the Bio-Rad Real-Time CFX96 PCR System, using precision melt super mix (Bio-Rad Laboratories, Hercules, CA, USA). Specific pairs of oligonucleotide primers (forward and reverse) were designed using Primer3 v4 (http://frodo.wi.mit.edu) to flank FBN-1 exons 14, 19, 28, and 42 and TGF-*β*R2 exon 6 (Table 1). PCR was performed in a 20 *μ*l reaction volume containing 10 *μ*M of each primer, 25 ng genomic DNA, and master mix high resolution. Cycling conditions were set as follows: an initial denaturation step of 95°C for 10 min, followed by 40 cycles of 95°C for 10 s, annealing *T* (according to each primer) for 30 s, and 72°C for 30 s. After completion of amplification, DNA was heated at 95°C for 30 s, kept at 50°C for 30 s, and then melted from 65 to 95°C (increment 0.2°C, dwell time 10 s). The results were analyzed using the Bio-Rad Precision Melt Analysis software. Melting profiles were normalized, grouped, and displayed as fluorescence-versus-temperature plots or subtractive difference plots (−df/dt vs. *T*). All samples with distinguished melting curves from wild type were confirmed by a duplicate study. Oligonucleotide primer sequences to carry out HRMA of the FBN-1 and TGF-*β*R2 coding regions are shown (Table 1). For the study of COL3A1, the molecular analysis was performed on genomic DNA by polymerase chain reaction amplification of all coding exons and the flanking intron regions. The amplicons were analyzed by the sequencing-by-synthesis (SBS) technology (MiSeq Personal Sequencer, Illumina, San Diego, California, United States). The gene panel including COL3A1, NOTCH1, MYH11, and SMAD3, but not all, was identified in these groups of patients. The presence of the variants was confirmed by Sanger sequencing.

### 2.15. Statistical Analysis

The SigmaPlot 14 program (Jendel Corporation, 1986-2017) was used to generate the graphs and to perform the statistical analyses. Statistical significance was determined by the Mann-Whitney rank sum test followed by the normality test (Shapiro-Wilk). The correlation was made with Spearman's test. The data are presented as the median and Min–Max range. Differences were considered statistically significant when *p* ≤ 0.05.

## 3. Results

### 3.1. General Characteristics

Out of the 10 LDS patients, 4 were men and 6 were women. The demographical characteristics of the patients are shown in Table 2.

### 3.2. Mutations in LDS Patients

The percentage of positive mutations considering the mutation in any of the 4 exons of FBN-1 was of 80%. It was 100% for TGF-*β*R2 and 10% for COL3A1. Also, 90% of the patients shared mutations in all genes.

### 3.3. Correlations

The correlations between the LPO and aortic diameters and TAC and aortic diameters are shown in Table 3.

### 3.4. General Variables

Table 4 shows the different general variables of both C subjects and patients with LDS. These are demographic variables and serum biochemistry; in this table, the results are expressed as median with the Min–Max values.

### 3.5. Enzymatic Activity and Expression

Table 5 shows the enzymatic activity and expression in the homogenate of the thoracic aortic aneurysms in both C subjects and patients with LDS.

### 3.6. Enzymatic Activity

**Figure 1:**
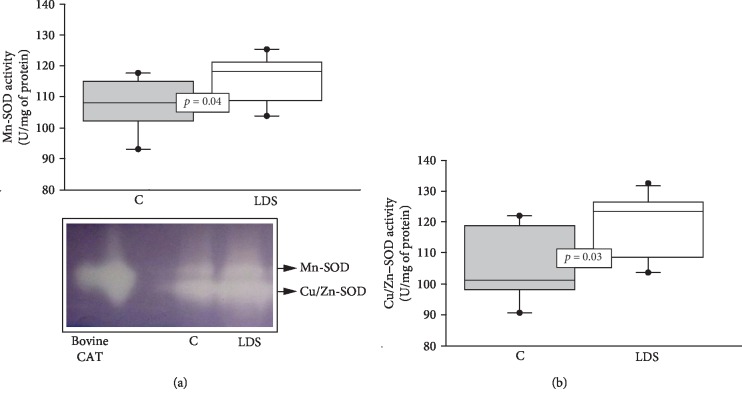
(a) Average activity of the Mn-SOD and (b) average activity of the Cu/Zn-SOD in LDS patients (*n* = 10) and C subjects (*n* = 9). Values expressed represent the median and Min–Max range. The image of the center is a representative gel of the electrophoresis of the SOD isoforms. Abbreviations: LDS = Loeys-Dietz syndrome; C = control subjects.

**Figure 2:**
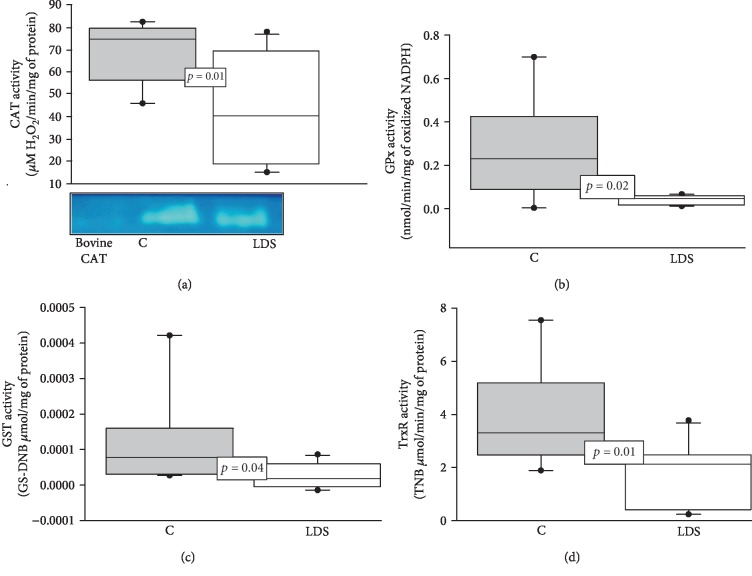
(a) Average activity of CAT. The image below the graph is a representative native gel of the electrophoresis. (b) GPx activity, (c) GST activity, and (d) TrxR activity in LDS patients (*n* = 10) and C subjects (*n* = 9). Values are expressed as the median and Min–Max range. Abbreviations: LDS = Loeys-Dietz syndrome; C = control subjects.

**Figure 3:**
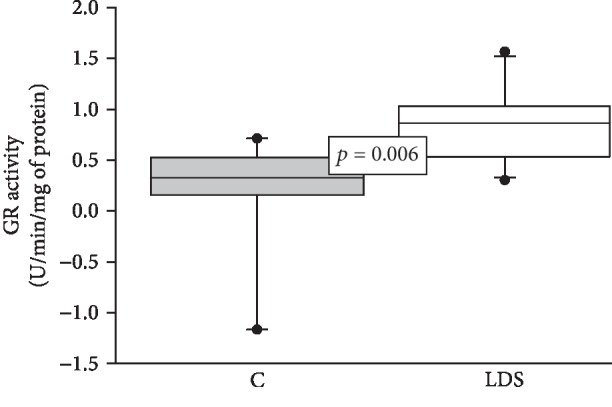
Average activity of GR in LDS patients (*n* = 10) and C subjects (*n* = 9). Values are expressed as the median and Min–Max range. Abbreviations: LDS = Loeys-Dietz syndrome; C = control subjects.

The Mn- and Cu/Zn-SOD isoforms showed a significant increase in their activities compared to the C group (*p* = 0.04 and *p* = 0.03, respectively) (Figures 1(a) and 1(b)). The enzymatic activities of CAT (*p* = 0.01), GPx (*p* = 0.02), GST (*p* = 0.04), and TrxR (*p* = 0.01) in patients with LDS were significantly decreased when compared to those in the C subjects (Figures 2(a)–2(d), respectively). The GR activity showed a significant increase (*p* = 0.006) in patients with LDS when compared to the C subjects (Figure 3).

### 3.7. eNOS, Cu/Zn-SOD, ORX, and Nrf2 Expressions

**Figure 4:**
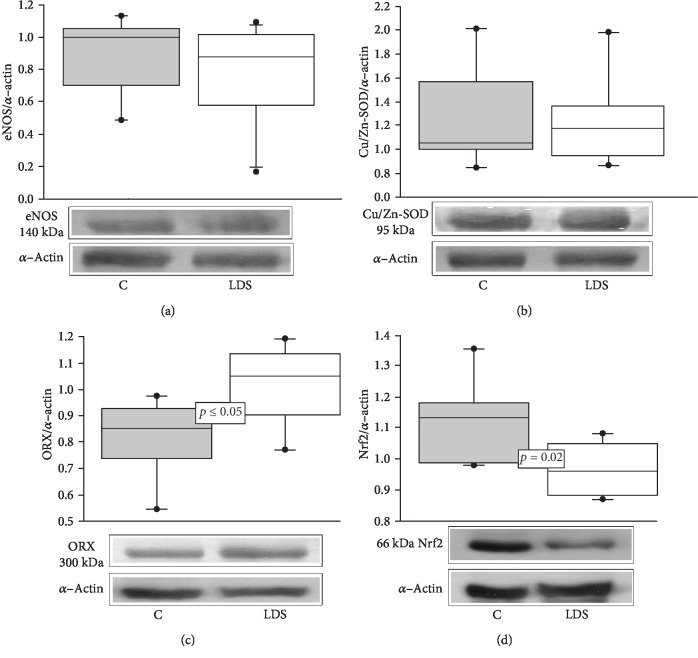
(a) Representative histograms of eNOS/*α*-actin expression. (b) Cu/Zn-SOD/*α*-actin expression, (c) ORX/*α*-actin expression, and (d) Nrf2/*α*-actin expression in LDS patients (*n* = 10) vs. C subjects (*n* = 9). Values are expressed as the median and Min–Max range. Abbreviations: LDS = Loeys-Dietz syndrome; C = control subjects.

The eNOS and Cu/Zn-SOD expressions did not show a significant difference in patients with LDS when compared to the C subjects (Figures 4(a) and 4(b)). The ORX and Nrf2 expressions showed significant increases and decreases, respectively, in patients with LDS when compared to the C subjects (*p* ≤ 0.05 and *p* = 0.02, respectively; Figures 4(c) and 4(d)).

### 3.8. Nonenzymatic Antioxidant System

The TAC levels, GSH, and vitamin C concentration showed a significant decrease (*p* = 0.006, *p* = 0.006, and *p* = 0.01, respectively) in patients with LDS when compared to the C subjects (Table 6). The protein carbonylation in patients with LDS showed a significant increase (*p* = 0.01, Table 6). The Se concentration in the TAA homogenate from patients with LDS was significantly decreased (*p* = 0.01, Table 6) when compared to that from the C subjects. However, the LPO index and NO_3_^−^/NO_2_^−^ ratio tended to rise without reaching significance in patients with LDS compared to the C subjects (Table 6).

### 3.9. Histology

#### 3.9.1. Hematoxylin-Eosin Staining

**Figure 5:**
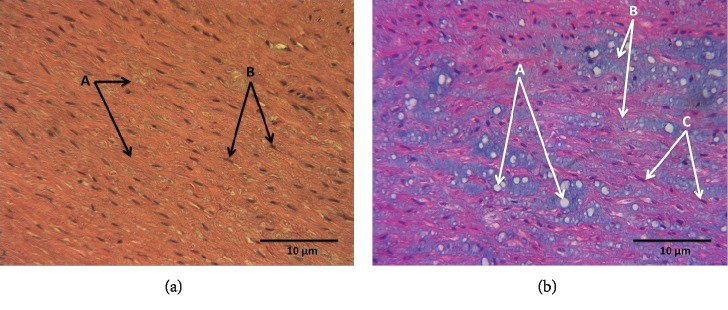
(a) Representative photomicrograph at 40x with hematoxylin-eosin staining of the aortic middle wall of a control subject. (A) Elastic fibers in deep pink. (B) Dark-colored fibrocyte nucleus. (b) Representative photomicrograph of the aortic middle wall of an LDS patient. (A) Cystic necrosis, with accumulation of amorphous material. (B) Elastic and collagen fibers in reddish and deep pink. (C) Blue-stained fibrocyte nucleus.

Figure 5(a) shows the representative photomicrograph of the aortic middle wall of a C subject, showing black nuclei of fibrocytes arranged in fascicles, red elastic fibers, and pink collagen fibers, without cystic necrosis.  Figure 5(b) shows the representative photomicrograph of the middle aortic wall of a patient with LDS, showing blue nuclei of fibrocytes arranged in fascicles, in red elastic fibers, and in pink collagen fibers; the presence of cystic necrosis is observed in white.

#### 3.9.2. Masson's Trichrome Stain

**Figure 6:**
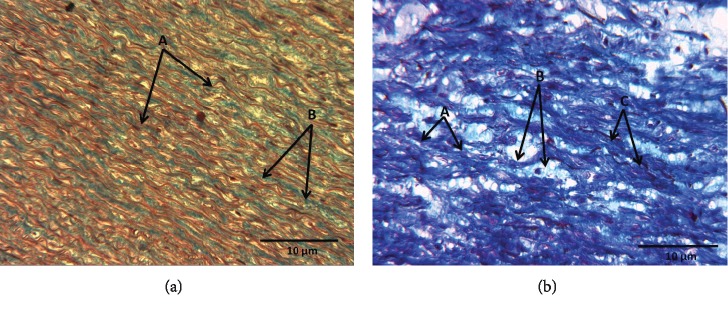
(a) Representative photomicrograph at 40x with Masson's trichrome staining of the aortic wall of a control subject. (A) Elastic fibers in red. (B) Collagen fibers in blue. (b) Representative photomicrograph of the aortic middle wall of an LDS patient. (A) Collagen fibers in deep blue color. (B) Cystic necrosis with the presence of amorphous material in faint blue color. (C) Elastic fibers in red color.

Figure 6(a) shows the representative photomicrograph of the aortic middle wall of a C subject; elastic fibers are observed in red, collagen fibers are observed in blue, and no cystic necrosis is observed.  Figure 6(b) shows the representative photomicrograph of the aortic middle wall of a patient with LDS; elastic fibers are observed in red, collagen fibers are observed in blue, and cystic necrosis is observed in white with the presence of amorphous material in faint blue.

#### 3.9.3. Weigert's Staining

**Figure 7:**
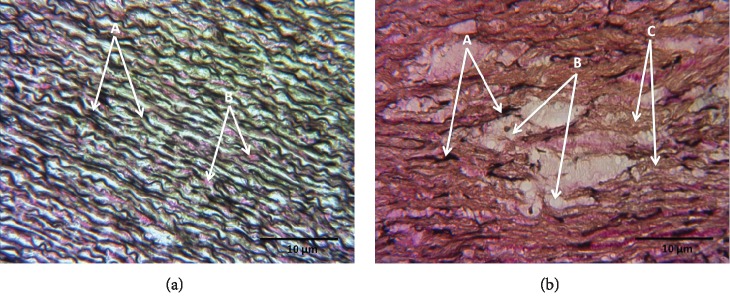
(a) Representative photomicrograph at 40x with Weigert's staining of the aortic wall of a control subject. (A) Elastic fibers in black color. (B) Collagen and elastic fibers in pink and light brown color. (b) Representative photomicrograph of the aortic wall of an LDS patient. (A) Elastic fibers. (B) Cystic necrosis. (C) Collagen fibers.

Figure 7(a) shows the representative photomicrograph of the aortic middle wall of a C subject; elastic fibers are observed in black, collagen fibers are observed in brown-yellow, and cystic necrosis is not distinguished.  Figure 7(b) shows the representative photomicrograph of the aortic middle wall of a patient with LDS; fragmented elastic fibers are observed in black, collagen fibers are observed in brown-yellow, and cystic necrosis is distinguished in white.

## 4. Discussion

In the MS, there is an imbalance of the redox homeostasis that contributes to the alteration and development of the TAA [33]. However, in LDS which is a rare variant of the MS [5], the role of the imbalance in the redox homeostasis has not been evaluated. The main LDS characteristic is the presence of TAA with rapid progression that decreases the survival of the patients [3]. Therefore, the aim of this work was to analyze the oxidant/antioxidant profile in the TAA from patients with LDS. An imbalance similar to that found in MS with a positive feedback oxidizing background was found.

### 4.1. The FBN-1, TGF-*β*R2, and COL3A1 Coding Regions

The molecular testing in LDS is crucial to assess the diagnosis and management of the patients. We analyzed the mutations in the FBN-1, TGF-*β*R2, and COL3A1 genes. The percentage of positive mutations considering the mutation in any of the 4 exons of FBN-1 was of 80%. It was 100% for TGF-*β*R2 and 10% for COL3A1. 90% of the patients also shared mutations in all genes. The fact that these mutations were not found in the FBN-1 gene in all patients is due to the size of the gene which is greater than 200 k. The coding sequence is divided into 65 exons, and more than 800 mutations are known within it. This makes the complete study of the gene unavailable and expensive. The same happens for the TGF-*β*R2 gene. Therefore, we only examined those exons that have the highest frequency in this disease and mutations in the FBN-1 gene.

Most investigations on LDS focus on the study of mutations [34]. Mutations in FBN-1 are associated with 60-90% of the diagnosis [35]. In addition, mutations in the genes that encode for TGF-*β*1 and TGF-*β*2 have been identified in patients having this disease. TGF*β* is involved in the development and maintenance of blood vessels and craniofacial growth [34]. In patients in whom no mutations in FBN-1 and TGF*β* were found, homozygous deletions in the COL3A1 gene have been identified, and these mutations lead to structural alterations of the collagen that could cause aortic dissection [36]. Mutations affecting the intracellular kinase domain of this protein can disturb TGF-*β* signaling, which subsequently leads to features of LDS patients. In fact, TGF-*β*R2 mutations have been related to skeletal abnormalities, aortic dilation, and ED. Mutations of the FBN-1 gene have also been found in MS, and they produce a deconstructed protein product which cannot bind to the latent TGF-*β*R1 [4, 9, 10]. Activation of the latent TGF-*β*R1 by ROS could be associated with the decrease in GPx activity [37]. Therefore, an increase in the TGF-*β*R1 concentration in the TAA could be expected, and an increase in the TGF-*β*R1 and TGF-*β*R2 receptor expressions could upregulate the noncanonical and canonical TGF-*β* pathways which are increased in myocytes from TAA [9].

TGF-*β*R1 and TGF-*β*R2 can upregulate the expression of elastases and metalloproteinases (MMPs), which may enhance elastin degradation. MMPs participate in the elastic fiber disintegration, which is observed in the representative microphotographs of the patients with LDS. In these patients, there is a reduction of connective tissue elasticity that results in weakness of the aneurysm aortic wall [9, 16, 38]. These breakings in structure could be associated with decreases in GSH and GPx. ROS can affect the migration, differentiation, and proliferation of the myocyte cells which express MMPs that may be altered by the GPx that is dynamically regulated in the blood vessels [39]. They are the result of the TGF-*β*R1 and TGF-*β*R2 increases and ROS accumulation that contribute to the cystic necrosis of the aortic wall, vasomotor dysfunction, and ED, thus favoring the aneurysm formation [6, 9].

### 4.2. Antioxidant Enzymatic Profile in TAA from Patients with LDS

Several studies in MS mouse models have described vasomotor dysfunction in the TAA associated with ROS overproduction. This has been related to alterations in antioxidant enzymes such as SOD isoforms [9, 18] and to several diseases such as Turner syndrome, arterial hypertension, and some MS variants [16]. However, another investigation has shown a decrease in SOD isoforms due to the increase in O_2_^−^ in the TAA in a mouse model of MS. This decrease was related to the deterioration of aortic function [10].

Our results suggest that the alteration of the activity of Cu/Zn- and Mn-SOD can be due to the overproduction of O_2_^−^ through ORX and NADPH oxidase overexpression in tissue from the TAA [10]. An increase in ORX expression could increase the O_2_^−^ concentration and stimulate Cu/Zn- and Mn-SOD activity, increasing the H_2_O_2_ concentration. This ROS is more harmful than others due to its capacity to diffuse through the cell membrane [40]. However, in our determinations, the expression of the Cu/Zn-SOD did not show significant changes. These results can be explained considering that the enzyme expression is not necessarily related to an increase in its activity since the expressed protein may be inactive or have a lower expression while having a greater activity. Thus, the degree of phosphorylation of the active site of SOD could increase its activity [41]. The increase in SOD activity in the patients with LDS suggests that the SOD isoforms try to counteract the O_2_^−^ overproduction. However, this seems counterproductive since it results in H_2_O_2_ overproduction.

CAT is responsible for the H_2_O_2_ elimination, and its overexpression can decrease OS in the TAA [12]. CAT activity is altered in aortas of patients with MS through continued H_2_O_2_ exposure [10]. Our results show that CAT activity is significantly decreased in TAA of patients with LDS. This may be due to an H_2_O_2_ overproduction by the SOD isoforms [42, 43]. Also, the excessive O_2_^−^ concentration can cause glucose-6-phosphate inhibition that catalyzes the formation of NADPH, which serves as an electron donor to CAT [44].

GPx is among the enzymes responsible for the H_2_O_2_ detoxification. It is more efficient than CAT when removing intracellular H_2_O_2_ and organic peroxides [15]. The GPx activity was decreased in patients with LDS, and this may be due to the low availability of GSH in these patients. The GPx has a Se atom in the form of selenocysteine in its catalytic site, and Se is a limiting factor of the antioxidant defense [45]. The deficiency of Se leads to cardiovascular pathologies [46] and diseases such as Kashin-Beck and Keshan [47–49]. In the patients with Keshan disease who have dilated cardiomyopathy, the Se deficiency decreases the GPx activity [49]. This suggests that selenoprotein expression or activity is regulated by the concentration of this trace element [50]. Our results show that the Se concentrations are decreased in patients with LDS, and this may impact on the activity of Se-dependent enzymes such as GPx and TrxR. Also, Se is absorbed in the form of organic compounds and in the presence of vitamins A, D, and E. Patients with LDS probably have an intestinal malabsorption syndrome which could cause the decrease in the Se [51]. Our results also showed a decrease in the concentration of vitamin C, which is important to regenerate vitamin E [15]. The decrease in both may affect the Se absorption.

GST is responsible for conjugating GSH with xenobiotics, peroxidized lipids, hydroperoxides, and H_2_O_2_ among other molecules favoring their elimination [15]. The substrates used by the GST are formed as a result of ROS modification of macromolecules [52]. A decrease in GST in aortas of patients with MS [14] and in patients with other pathologies that affect the cardiovascular system has been associated with OS and with an increase in LPO [16]. Our results show that the GST activity is decreased in patients with LDS. This suggests that the GST activity is compromised by the low availability of GSH. This contributes to the tendency to increase LPO. In addition, the increase in protein carbonylation may depend on the decrease in the TrxR activity, which would be reflected in a smaller reduction of thiol groups in proteins. TrxR is another Se-dependent protein possessing a selenocysteine in its catalytic site [53]. OS induces a compensatory increase in TrxR to reduce the oxidative modification of proteins present in several pathologies [53]. However, TrxR is decreased in pathologies with severe OS and metabolic disturbances [54]. The low TrxR activity in patients with LDS could increase the carbonylation and be associated with the OS present in this syndrome.

### 4.3. Antioxidant Nonenzymatic Profile in TAA from LDS Patients: Glutathione Levels and Glutathione Reductase

GSH is the main defense mechanism against ROS and electrophiles [55]. It is formed by *γ*-*ι*-glutamyl-*ι*-cysteinyl-glycine. GSH can also be a substrate for metabolic pathways such as the one mediated by the SOD isoforms and ORX. GSH homeostasis occurs mainly through the balance of consumption and supply [34]. When GSH reduces target molecules, electrophiles, or ROS, the thiol group of the cysteine is oxidized through the GPx activity. Excessive GSH oxidation by GPx might be due to chronic OS in patients with LDS. GSH is then reduced to GSSG by GR to restore GSH levels [55, 56]. GR requires NADPH consumption for its activity [40, 56]. A decrease in the GR activity will cause a decrease in the reduced GSH concentrations resulting in an increase in the level of the ROS. In several pathological processes, an alteration of the GR activity and in the GSH levels has been found, which is associated with an increased risk of OS [57]. Our results show that the GR activity was increased in patients with LDS, but despite this, the GSH values were not restored. This may be due to low levels of GSH which may stimulate the GR activity [58]. A low GSH level limits the activities of GPx and GST, and in turn, the low activities of GPX and GST may contribute less to the scavenging of H_2_O_2_ and O_2_^−^ and this can favor and contribute to chronic OS in patients with LDS. It may also be due to a compensatory protective mechanism of the cells against oxidant agents [59]. Moreover, the GSH produced by GR may be oxidized and therefore be insufficient to compensate for the high production of ROS [60].

Another possible cause of the GSH decrease could be the alteration of the enzyme *γ*-glutamyl-cysteine synthase which is responsible for the GSH synthesis or for a deficient amount of the amino acids that constitute GSH [56]. In addition, the TAC results which include the GSH level show a decrease in the LDS patients. This confirms the presence of OS in the TAA of tissues from patients with this disease. Likewise, the LPO index showed a tendency to increase, and this could lead to an increase in prooxidant compounds such as 4-hydroxinonenal and MDA [61].

### 4.4. Expressions of Nrf2, Xanthine Oxidase, and Endothelial Nitric Oxide Synthase

The Nrf2 transcription factor is responsible for the regulation of the inducible expression of numerous antioxidant enzyme genes such as GPx, SOD isoforms, GST, and TrxR [62]. The low levels of Nrf2 expression in patients with LDS suggest that it can be responsible, in part, for the decrease in the activity of the antioxidant enzymes. Our results showed a tendency to decrease eNOS expression in the patients with LDS. Other studies have shown that the iNOS activity is increased and the activity of eNOS is decreased in the homogenate of TAA from patients with MS [63]. This result also suggests that the NO production mostly comes from the iNOS pathway and to a lesser extent from the eNOS [39]. Furthermore, in MS animal models, O_2_^−^ overproduction by the NADPH oxidase and ORX pathway is associated with increase in NO production by iNOS and is involved in inflammatory processes [14, 18, 63]. This promotes the ONOO^−^ formation that contributes to OS in the TAA [64]. High concentrations of ONOO^−^ can lead to apoptosis in endothelial cells and may favor the activation of MMPs 1 and 2 in vascular smooth muscle cells degrading essential proteins to maintain the anatomical integrity of the aorta [64].

### 4.5. Histological Characteristics

The observation of the histological cuts from the TAA segments of patients with LDS showed cystic necrosis and accumulation of amorphous material. This suggests the development of fibrosis in the TAA that may be associated with chronic OS. The participation ROS in the formation of the abdominal aortic aneurysm was associated with the overexpression of the MMPs 2 and 9 [65]. The results with Weigert's staining showed fragmentation, thickening, and a smaller number of elastic fibers in the LDS group. This could be related to aortic stiffness. The elastic fiber fragmentation in the aorta is related to the interaction between OS, MMPs, and the TGF-*β* deregulation [66, 70]. Our results show a collagen increase in photomicrographs from the patients with LDS. This is probably due to deregulation in TGF-*β* signaling.

## 5. Conclusion

The antioxidant enzyme activities, including those of SOD isoforms, CAT, TrxR, and GST, decrease in the TAA of patients with LDS. These decreases in the enzyme activities favor the accumulation of ROS that contributes to GSH decrease and favor LPO and carbonylation. The decrease in Se and Nrf2 also impacts on the activity and/or expression of some of these antioxidant enzymes. The GR increase does not completely restore the GSH concentration, which is reflected in the decrease in the TAC and in the enzymes that use it which contributes to and favors ROS production. This leads to a positive feedback oxidizing background which contributes to the TAA formation.

### 5.1. Perspectives

The goal of the medical treatment in LDS is to delay the progression of aortic dilation to avoid catastrophic complications. This study proposes that the use of antioxidants together with the current treatments could help patients with LDS. The use of *β* blockers, as well as the use of angiotensin 1 antagonists, constitutes the first-line therapy to this day for LSD and MS [67]. However, the scientific evidence supporting these therapies is limited since it has been obtained from simple randomized trials with a restricted sample number [68]. In 2007, a meta-analysis of 6 studies that included 802 patients with MS showed no difference in the results of *β* blocker therapy in relation to mortality and morbidity [69].

The excessive activity of MMPs resulting in an excessive deposition of matrix elements such as proteoglycans and collagen which are organized in a random manner and fragmentation of elastic fibers is similar to that seen in atheromatous plaques, and therefore, the use of statins such as pravastatin at a dose of 40 mg/day might also be useful for the treatment of TAA in LDS and MS. Statins are inhibitors of HMG-CoA reductase and are used as CT-reducing agents with proven efficacy to reduce morbidity. Moreover, a study concluded that statins could have a similar effect to that of losartan in their capability to attenuate aortic root dilation in a mouse model with MS. Although these drugs reduced excessive protein manufacturing by vascular smooth muscle cells [70], inhibition of MMPs by these drugs may alter fibrillin-1 which is the component of the microfibrillins that provides a connection between elastin fibers and vascular smooth muscle cells [70, 71]. The structural homology between fibrillins and latent TGF-*β* binding proteins suggests an additional role for FBN-1 to direct TGF-*β* to the extracellular matrix and to keep it in an inactive state. Therefore, an FBN-1 deficiency would lead to excessive TGF-*β* signaling [71] which would not benefit LDS patients.

Deregulation of TGF-*β* signaling contributes to aortic and mitral valve pathology in mouse models with MS [72]. This is supported by the observation of a Marfan phenotype in humans with mutations in the TGF-*β* receptor [73]. Fibrillin-1 deficiency that leads to deregulation of TGF-*β* signaling causes vascular smooth muscle cells to reshape the extracellular matrix through the SMAD pathway [73]. However, other mechanisms such as redox status also importantly participate in the expansion of TAA in LDS together with the genetic predisposition, according to our findings, and therefore, therapeutics in this regard should be evaluated.

It is a very important state that during the evolution and progression of aneurysms, patients are typically asymptomatic and it is not until aortic dissection occurs, generating severe pain, that the doctor suspects the diagnosis. At that stage of the disease, it is too late for many therapeutic interventions and sudden death can happen in up to 50% of patients. Since it is clear that OS plays an important role in the development of aneurysms, OS should be monitored in clinical practice and aimed during treatment since diagnosis. The execution of randomized clinical trials in this regard should be addressed. Finally, it is possible that the use of a combination of antioxidants or antioxidants together with AT-1 inhibitors and statins may have an impact on the control of aortic dilation. Their beneficial effect could be monitored by various avant-garde imaging studies. In this sense, the treatment with antioxidant supplements such as N-acetylcysteine, a GSH donor, vitamins C and E, or combination of these or other antioxidants could contribute to the deceleration of the aggressive and lethal development of the TAA and increase the quality of life and survival in patients with LDS.

### 5.2. Limitations

The main limitation of this study was the obtainment of TAA fragments from patients with LDS. As described above, this disease is a variant of MS with unknown incidence that may be of around 1/10000. Only 10 patient samples could be obtained in a period of approximately 9 years. Another limitation was the improbability of having matched C subjects for gender and age. Although may there be a correlation of TAC with age, it is not possible to obtain aortic tissue from healthy subjects at specific ages. The only way to obtain these samples is finding subjects having a surgical indication where there is a possibility to ethically withdraw a small sample. This depends on the treatment applied and/or the surgical technique employed and on the obtainment of their informed consent. However, even if there are age differences in C subjects and patients with LDS, there is certainty that there were no comorbidities or aortic damage as shown in the photomicrographs of the C subjects.

## Figures and Tables

**Table 1:** Oligonucleotide primer sequences to the FBN-1 and TGF-*β*R2 coding regions.

Gene	Exon	Forward primer	Reverse primer	Annealing temperature (°C)
FBN-1	14	5′-TGGCCGGATCTGCAATAATG-3′	5′-ACAGTTCTTCCCATCTCGTGT-3′	56
FBN-1	19	5′-TGGTGCAGATATAAATGAATGTGC-3′	5′-GAAAATGGGTAAAACTTCTCACCA-3′	64
FBN-1	28	5′-AGATCCTCTCCTATGCCGAG-3′	5′-GATACACGCGGAGATGTTGG-3′	57
FBN-1	42	5′-GCATCACCAACCCTCCAATC-3′	5′-CACCTGTACTTGGGATGGGA-3′	56
TGF-*β*R2	6	5′-ATGGGCCTCACTGTCTGTTT-3′	5′-CACAATGATGCTGGTCCACA-3′	55

**Table 2:** Demographic characteristics of Loeys-Dietz syndrome patients.

Sex	Age	MS family history	Clinical characteristics	Gene	Dao (mm)	*Z* score	Surgery type	Evolution over time
M	15	The father died at age 26 and was diagnosed with LDS	Dolichocephaly, malar hypoplasia, retrognathia, ogival palate, enlarged uvula, hypertelorism, low implantation of atrial pavilion, milia in the malar region, arm stroke ratio size > 1.06, stretch marks, pectus carinatum, scoliosis, equine foot varus. Steinberg and Walker-Murdoch positive, without ectopia lentis	FBN-1 exon 28 and TGF-*β*R2 exon 6	29	4.07	Aortic root replacement by David's procedure (09/06/2013)	He died a year after the cardiovascular postoperative period, due to an acute cholecystitis complication
F	22	The father and sister died at 27 and 17 years ago with LDS diagnosis	Hypertelorism, ogival-type palate, dental overlap, uvula, widened millia, pectus carinatum, scoliosis, Steinberg and Walker-Murdoch, positive flat feet and valgus. Arm/height atio rgreater than 1.04, without ectopia lentis	FBN-1 exon 15 and TGF-*β*R2 exon 6	48	5.2	Aortic root replacement by David's procedure (02/07/2013)	Satisfactory evolution currently in functional class 1 (NYHA) was evaluated in consultation in May 2019
M	13	His mother was diagnosed with LDS, died at 22 due to aortic rupture, and he has a sister with LDS	Dolichocephaly, hypertelorism, ogival palate, uvula bifida, micrognathia, low implantation of atrial pavilion, pectus carinatum of right hemithorax (asymmetric), stretch marks, Steinberg sign and Walker-Murdoch positive, flat foot, contractures in eyes, scoliosis, and mitral valve prolapse	TGF-*β*R2 exon 6 and COL3A1	24	2.98	Aortic surgery by David's procedure and he had mitral plasty (11/01/2013)	Since 11/11/2013, he has had dyskinetic and ballism that improved with anticonvulsive, the CT scan did not show aneurysms, he is hemodynamically stable, and he remains alive until 2019
F	18	No history of MS or LDS	She showed dolichocephaly, hypertelorism, micrognathia, ogival palate and overlap, dental bifida tooth, arm reaction/height > 1.06 hyperelasticity, pectus carinatum, contractural arachnodactyly in feet and hands, flatfoot and varus Steinberg- and Walker Murdoch-positive scoliosis ecstasy. In her study of magnetic resonance, she had artificial tortuosity	FBN-I exon 28 and TGF-*β*R2 exon 6	46	9.5	Aortic surgery by David's procedure with a 30 mm graft (04/10/2012)	Stable evolution, functional class 1. Last consultation July 2019
M	38	Mother, one brother, and 7 cousins with MS, 1 cousin with LDS	Hypertelorism, ogival palate, dental overlap, bifida uvula, scoliosis, stretch marks. Without lens dislocation, size and stroke 1.98 and height 1.90	FBN-1 exon 60+TGF-*β*R2 exon 6	48	NA	Aortic surgery by David's procedure (20/01/2014)	Hemodynamically asymptomatically stable. In 2019, it was found in functional class 1
F	14	Father with LDS	Hypertelorism, uvula bifid, scoliosis pectus carinatum	FBN-1 exon 28 and TGF-*β*R2 exon 6	54	4.30	Aortic surgery by Bentall and De Bono (17/12/2018)	In 2019, he went to evaluation and was stable
F	21	The background is unknown	Hypertelorism, ogival palate, wide uvula, pectus carinatum valgus, foot without lens dislocation. Tortuous arteries	TGF-*β*R2 exon 6	25	3.47	Aortic surgery by David's procedure (07/09/2015)	Satisfactory evolution. The last revision was in 2019
M	46	The background is unknown	With hypertelorism, uvula bifida, scoliosis, flat feet	FBN-1 exon 28 and TGF-*β*R2 exon 6	63	NA	Aortic surgery by Bentall and De Bono (09/09/2015)	He came to the office until 2019
F	25	Her father died of acute aortic dissection at age 24 and was diagnosed with MS	Hypertelorism, millia in the malar region, pectus carinatum, normal uvula flat foot. By imaging, she had tortuous arteries	FBN-1 exon 42 and TGF-*β*R2 exon 6	46	NA	Aortic surgery by Bentall and De Bono (18/12/2015)	She came to the hospital in 2019, and she was stable
F	35	The background is unknown	Hypertelorism, pectus carinatum, flat feet, tortuous arteries. Height 1.59 weight 64	FBN-1 exon 28 and TGF-*β*R2 exon 6	58	NA	Aortic surgery by Bentall and De Bono (06/04/2017)	She visited the hospital in 2019

Abbreviations: M = male; F = female; NYHA: New York Health Association; MS = Marfan syndrome; LDS = Loeys-Dietz syndrome; Dao = diameter aortic.

**Table 3:** Correlations of LPO with TAC and aortic diameters.

	Total		C		LDS	
	*R* ^2^	*p*	*R* ^2^	*p*	*R* ^2^	*p*
LPO/TAC	-0.59	0.008	-0.55	NS	-0.45	NS
Carbonylation/TAC	-0.55	0.01	-0.49	NS	-0.32	NS
VBD/LPO	-0.01	0.97	0.40	NS	-0.75	NS
SUD/LPO	0.07	0.83	0.02	NS	-0.52	NS
VBD/TAC	-0.02	0.93	-0.47	NS	0.74	NS
SUD/TAC	-0.14	0.69	-0.29	NS	0.82	NS

Abbreviations: LDS = Loeys-Dietz syndrome; LPO = lipid peroxidation; TAC = total activity antioxidant; VBD = Valsalva breast diameter; SUD = sinotubular union diameter.

**Table 4:** Demographic variables and serum biochemistry in C subjects and patients with LDS.

	C Median (Min–Max)	LDS Median (Min–Max)	*p*
General characteristics			
Age (range)	62 (37–77)	23 (14–46)	0.001
Size (m)	1.65 (1.45–1.76)	1.67 (1.58–1.77)	NS
Weight (kg)	67 (56–105)	57 (33–71.5)	0.03
BMI (range)	27.8 (22.2–36.3)	20.8 (12.7 – 25.3)	0.005
Serum biochemistry			
CT (mg/dl)	125 (102–191)	129.5 (33–171)	NS
HDL (mg/dl)	39 (32–54)	48 (8.4–61)	NS
LDL (mg/dl)	73.5 (50–143)	84 (11.4–122)	NS
TG (mg/dl)	97.5 (67–148)	69 (57–118)	0.09

Abbreviations: BMI=body mass index; CT = cholesterol; HDL = high-density lipoproteins; LDL = low-density lipoproteins; TG = triglycerides.

**Table 5:** Enzymatic activity and expression in the homogenate of the thoracic aortic aneurysms in both C subjects and patients with LDS.

	C Median (Min–Max)	LDS Median (Min-Max)	*p*
Enzymatic activity			
Mn-SOD (U/mg/protein)	107.9 (93.3–117.8)	118.5 (103.9–125.2)	0.04
Zn/Cu-SOD (U/mg protein)	101.2 (90.9–122)	123.4 (103.5–132.23)	0.03
CAT (*μ*M/mg/protein)	74.7 (45.9–82.4)	40.2 (14.9–77.5)	0.01
GPx (nmol/mg/protein)	0.23 (0.003–0.69)	0.04 (0.01–0.06)	0.02
GST (*μ*M/mg/protein)	7 × 10^−5^ (2 × 10^−5^–4 × ^10−4^)	1 × 10^−5^ (1 × 10^−4^–8 × 10^−5^)	0.04
TRx (*μ*M/mg/protein)	3.30 (1.8–7.5)	2.1 (0.23–3.7)	0.01
GR (*μ*M/mg/protein)	0.32 (-1.1–0.71)	0.86 (0.30–1.56)	0.006
Enzymatic expression			
eNOS	0.99 (0.48–1.13)	0.87 (0.17–1.08)	NS
Nrf2	1.13 (0.98–1.35)	0.95 (0.87–1.08)	0.02
ORX	0.85 (0.54–0.97)	1.00 (0.77–1.19)	0.05

Mn-SOD = manganese superoxide dismutase; Zn/Cu-SOD = zinc copper superoxide dismutase; CAT = catalase; GPx = glutathione peroxidase; GST = glutathione reductase; TRx = thioredoxin reductase; GR = glutathione reductase; eNOS = endothelial nitric oxide synthase; Nrf2 = nuclear factor erythroid 2-related factor 2; ORX = xanthine reductase.

**Table 6:** Redox biomarkers of the nonenzymatic system in the homogenate of the thoracic aortic aneurysm patients with LDS and C subjects.

Parameters (mg/protein)	C Median (Min–Max)	LDS Median (Min–Max)	*p*
TAC (nmol Trolox)	119.6 (35–220)	15.09 (0.38–145)	0.006
LPO index (nmol MDA)	2.4 (0.53–7.4)	5.7 (2.3–15)	0.04
Carbonylation (ng carbonyls)	5.5 (2.04–11.8)	10.4 (4.3–21.1)	0.01
GSH (nM)	9 × 10^−2^ (1 × 10^−3^–6 × 10^−2^)	8 × 10^−3^ (3 × 10^−3^–1 × 10^−2^)	0.006
NO_3_^−^/NO_2_^−^ (nM)	2.6 (1.97–4.3)	3.37 (2.14–7.8)	NS
Vitamin C (*μ*M)	0.03 (0.02–0.04)	0.02 (0.01–0.04)	0.01
Se (pg)	119.6 (35–220)	15.09 (0.38–145)	0.01

Abbreviations: TAC: total antioxidant capacity; LPO: lipid peroxidation; GSH: glutathione; Se: selenium; LDS: Loeys-Dietz syndrome; C: control subjects. The data are presented as the median and Min–Max range.

## Data Availability

The datasets generated and analyzed during the current study are available from the corresponding author on reasonable request.
